# Extracellular vesicles derived from human adipose-derived stem cells promote the exogenous angiogenesis of fat grafts via the let-7/AGO1/VEGF signalling pathway

**DOI:** 10.1038/s41598-020-62140-6

**Published:** 2020-03-24

**Authors:** Yuanzheng Zhu, Jing Zhang, Xuan Hu, Zhaohui Wang, Shu Wu, Yangyan Yi

**Affiliations:** Department of Plastic Surgery, The Second Affiliated Hospital of Nanchang University, NO.1 of Minde Road of Nanchang, Jiangxi, 330006 P. R. China

**Keywords:** Cell signalling, Mesenchymal stem cells, Regeneration

## Abstract

Extracellular vesicles (EVs) derived from human adipose-derived stem cells (hADSCs) possess the proangiogenic potential for ischaemic diseases. Thus, our study aimed to evaluate the therapeutic effects of hADSC-EVs on fat grafting and explore the mechanism of hADSC-EVs promoting angiogenesis. The EVs released by hADSCs incubated under normal or hypoxic conditions were employed to supplement fat grafting in a nude mouse model. The proliferation, migration, tube formation and vascular endothelial growth factor (VEGF) secretion of vascular endothelial cells co-cultured with two kinds of hADSC-EVs were analysed. MicroRNA sequencing was performed to reveal the species and content of microRNAs in hADSC-EVs, the key microRNAs were blocked, and their effect in promoting angiogenesis was detected via above protocols as a reverse proof. The results demonstrate that hADSC-EVs could improve the survival of fat grafts by promoting exogenous angiogenesis and enhance the proliferation, migration, tube formation and VEGF secretion of vascular endothelial cells. In addition, the pro-angiogenic effect of hADSC-EVs *in vivo and vitro* could be enhanced by hypoxic pre-treatment. We found that the let-7 family, a kind of hypoxic-related microRNA, is enriched in hypoxic hADSC-EVs that contribute to angiogenesis via the let-7/argonaute 1 (AGO1)/VEGF signalling pathway.

## Introduction

Fat grafting has been widely used for soft tissue filling in cosmetic plastic surgery (such as breast augmentation and facial rejuvenation) and reconstruction surgery due to several diseases (such as soft tissue defects caused by facial muscle atrophy, tumour excision and breast reconstruction after breast cancer surgery)^1^. The technology of fat grafting has made significant advances in the past decades, being considered less technically challenging because of the sophisticated liposuction technique, low donor site morbidity after transplantation and reduced host immune responses^1–3^. However, the main limitation is the necrosis and absorption of grafts due to poor revascularization^4,5^. Therefore, the survival of fat grafts can still be improved.

The process of liposuction and injection leads to devascularisation and ischaemic injury of the adipose tissue. Since the adipose tissue is less tolerant to ischaemia^3^, early revascularization of the transplanted tissue is critical for the survival of fat grafts^4^. Various studies have demonstrated the promotion of angiogenesis by mesenchymal stem/stromal cells (MSCs). Yoshimura *et al*.^6^ developed a cell-assisted lipotransfer (CAL) strategy to co-transplant autologous adipose tissue with adipose stromal cells. The meta-analysis performed by Laloze *et al*.^7^ demonstrated that the CAL technology can significantly improve survival from 44 to 64%. With the progress on the research of the biological functions of MSCs, for fat grafting, the paracrine effect of adipose-derived stem cells (ADSCs) play important roles in angiogenesis and tissue regeneration^8^. In addition, studies in recent years have proposed that^9–11^ stem cells may have stronger paracrine functions under stress conditions, such as ischaemia and hypoxia, to promote the repair of ischaemic and hypoxic tissues.

Extracellular vesicles (EVs) are a kind of membrane vesicles released from cells into the extracellular environment that participate in intercellular communication^12^. There is evidence that in addition to MSC-derived cytokines, MSC-derived EVs, including exosomes and micro-vesicles, account for the proangiogenic potential of stem cells^13,14^. Moreover, Zhang *et al*.^15^ demonstrated that hypoxic treatment can enhance the pro-angiogenic effects of umbilical cord-derived MSCs to improve blood flow recovery in a rat hindlimb ischaemia model. However, why hypoxic treatment enhances the angiogenesis-promoting effect of MSC-EVs remains unclear. EVs transport a variety of biological substances, such as proteins, lipids, mRNAs, and microRNAs, among which microRNAs have been confirmed to be involved in the regulation of cell signalling pathways by intervention of RNA transcription^16,17^. Thus, this study aimed to demonstrate the effect of hypoxia-induced extracellular vesicles derived from human adipose-derived stem cells (hADSC-EVs) in promoting graft survival in a mouse model and the role of key microRNAs in promoting angiogenesis under hypoxic conditions.

## Results

### Characterisation of hADSCs and hADSC-EVs

**Figure 1:**
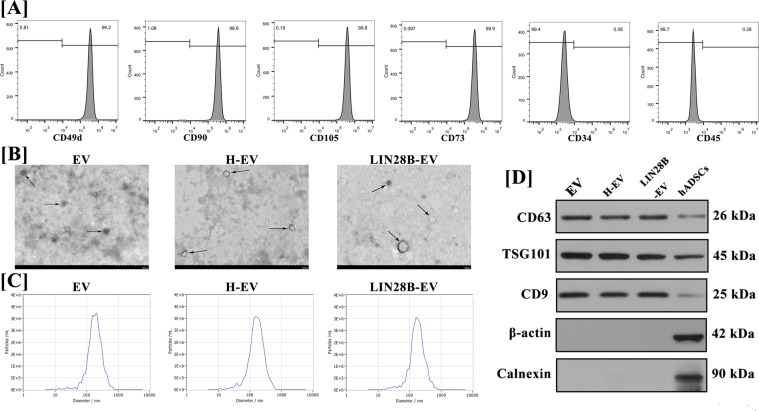
Characterisation of hADSCs and hADSC-EVs. (**A**) The expression of hADSCs surface markers CD49d, CD34, CD45, CD90, CD105 and CD73 was measured by flow cytometry. hADSCs were highly positive for CD105, CD90, CD73 and CD49d but negative for CD34 and CD45. (**B**) The size and the spheroid morphology of hADSC-EVs, hypoxic hADSC-EVs and LIN28B transfected hADSC-EVs (pointed by Black arrows) were shown under TEM (×25,000; 80 kV); scale bar: 500 nm. (**C**) The particle size of hADSC-EVs was assessed by NanoSight. NanoSight indicated that the average particle sizes of normal, hypoxic and LIN28B transfected hADSC-EVs were 183.1 ± 15.3 nm, 180.7 ± 14.9 nm and 174.5 ± 17.8 nm. (**D**) Western blot analysis indicated that hADSC-EVs had high expression of the surface markers of EVs, including CD63, CD9 and TSG101, which were relatively lower expressed in hADSCs. hADSC-EVs did not express β-actin and calnexin, which were higher expressed in hADSCs.

Flow cytometry analysis was performed to confirm that third-generation hADSCs expressed high levels of 49d, CD90, CD105 and CD73 and did not express CD34 or CD45 **(**Fig. 1A**)**. The EVs extracted from the supernatant of hADSCs cultured under hypoxic or normal conditions or LIN28B transfection exhibited few differences by the detection of transmission electron microscopy (TEM), NanoSight and western blot. Under TEM (×25000, 80.0 kV), the majority of hADSC-EVs were circular, bilayer membrane vesicles with different sizes **(**Fig. 1B**)**. NanoSight indicated that the average particle sizes of normal, hypoxic and LIN28B transfected hADSC-EVs were 183.1 ± 15.3 nm, 180.7 ± 14.9 nm and 174.5 ± 17.8 nm. (Fig. 1C**)**. Western blot analysis indicated that hADSC-EVs had high expression of the surface markers of EVs, including CD63, CD9 and TSG101, which were relatively lower expressed in hADSCs. hADSC-EVs did not express β-actin and calnexin, which were higher expressed in hADSCs **(**Fig. 1D**)**.

### hADSC-EVs improved the survival of fat grafts

**Figure 2:**
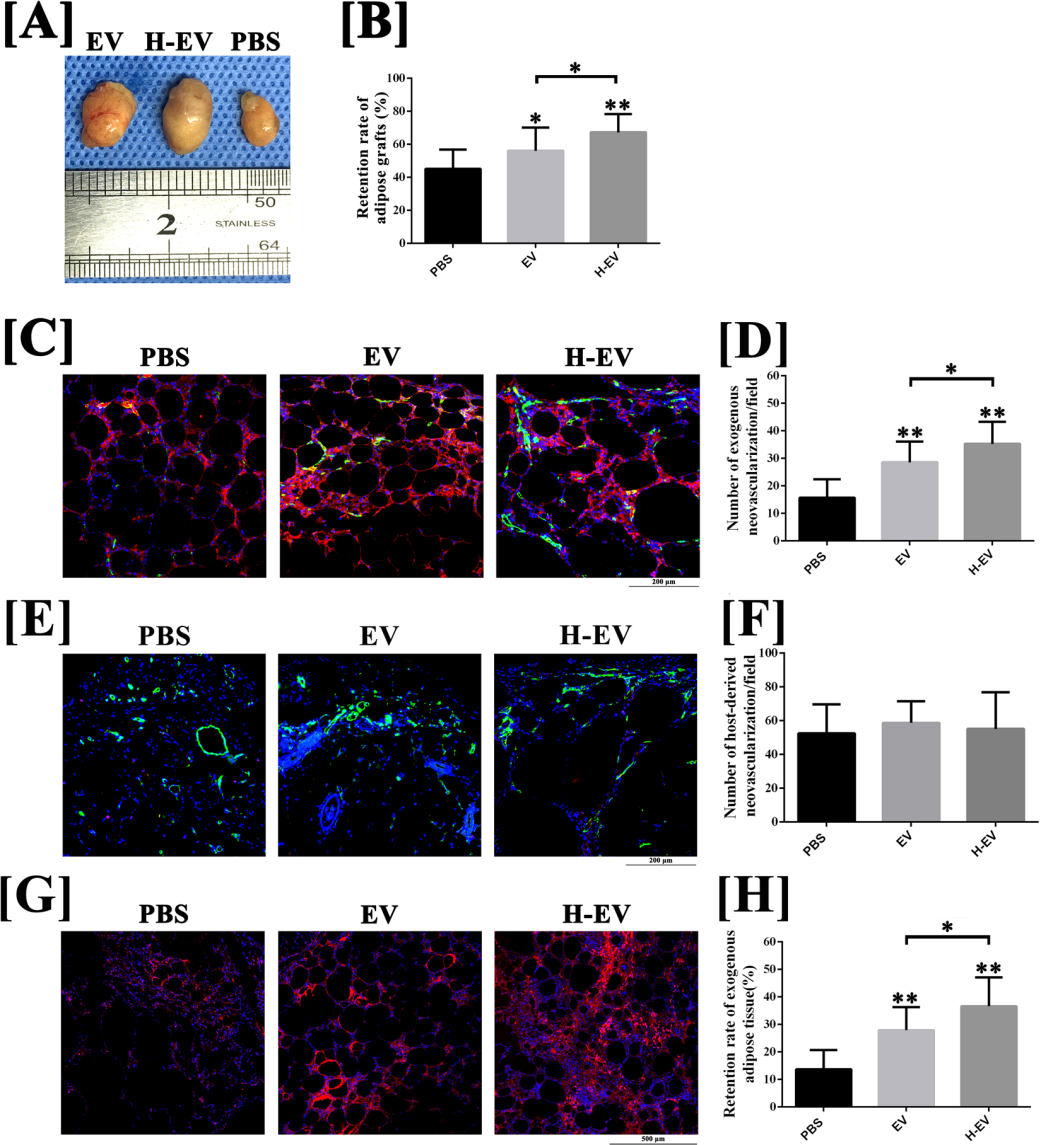
Animal model of fat grafting (n = 12). (**A**) The fat grafts harvested at post-grafted week 12. (**B**) Line charts were plotted to compare the volume retention rate of fat grafts at post-grafted week 12. (**C,D**); The results of CD31(green)/human mitochondria immunofluorescence(red) staining indicated that, at week 4 post-transplantation, increased exogenous angiogenesis was observed in fat grafts co-transplanted with hADSC-EVs and hypoxic hADSC-EVs, compared with those co-transplanted with PBS. Moreover, co-transplanting with hypoxic hADSC-EVs can further enhance the exogenous angiogenesis in the early stage, compared with co-transplanting with normal hADSC-EVs. Scale bar: 200 μm. (**E,F**) The results of immunofluorescence staining (CD31/human mitochondria) indicated that host-derived CD31-positive vessels (human mitochondria-) were observed in the peripheral region of fat grafts. Semi-quantitative analysis for the calculation of host-derived neovascularisation indicated that co-transplantation with hADSC-EVs or hypoxic hADSC-EVs exerted no significant effect on host-derived angiogenesis at post-grafted week 4, compared with those co-transplanted with PBS. Scale bar: 200 μm. (**G,H**) The results of immunofluorescence staining (human mitochondria) indicated that, at week 12 post-transplantation, significant increases in retention rate of exogenous adipose tissue were observed in grafts co-transplanted with hADSC-EVs and hypoxic hADSC-EVs, compared with that co-transplanted with PBS. The retention rate of exogenous adipose tissue can be further improved by co-transplanted hypoxic hADSC-EVs compared with normal hADSC-EVs. Scale bar: 500 μm. Data are presented as the mean ± SD (n = 12). *P < 0.05, **P < 0.01.

To evaluate whether hADSC-EVs can improve the survival of fat grafts, a nude mouse model of subcutaneous fat grafting was used. No mice died during the experiment. As shown in Fig. 2A,B, at week 12 post-transplantation, the volume retention rate of grafts co-transplanted with hADSC-EVs (56.0 ± 14.1%) or hypoxic hADSC-EVs (69.4 ± 9.5%) were significantly improved compared with those co-transplanted with phosphate-buffered saline (PBS, 35.8 ± 10.0%) (P < 0.05). In addition, co-transplanting with hypoxic hADSC-EVs can further improve the mean volume of fat grafts, compared with co-transplanting with normal hADSC-EVs (P < 0.05). However, no significant difference was observed in volume at week 4 (P > 0.05).

### hADSCs-EVs improved the survival of fat grafts by promoting exogenous angiogenesis

As shown in Fig. 2C,D, at week 4 post-transplantation, an increased number of CD31-positive vessels were observed in the exogenous region (human mitochondria+) of fat grafts co-transplanted with hADSC-EVs (28.5 ± 7.6/field) and hypoxic hADSC-EVs (35.2 ± 8.0/field), compared with those co-transplanted with PBS (15.7 ± 6.7/field) (P < 0.05). Co-transplanting with hypoxic hADSC-EVs can further enhance the exogenous angiogenesis in the early stage, compared with co-transplanting with normal hADSC-EVs (P < 0.05). However, as shown in Fig. 2E,F, no significant difference was observed in the calculation of host-derived vessels (P > 0.05). Furthermore, of note in Fig. 2G,H, at week 12 post-transplantation, significant increases in the retention rates of exogenous adipose tissue were observed in grafts co-transplanted with hADSC-EVs (27.9 ± 8.3%) and hypoxic hADSC-EVs (36.6 ± 10.5%), compared with those co-transplanted with PBS (13.6 ± 7.0%) (P < 0.05). The retention rates of exogenous adipose tissue can be further improved by co-transplantation with hypoxic hADSC-EVs compared with normal hADSC-EVs (P < 0.05). The above histological results suggest that hADSC-EVs possess an exogenous pro-angiogenic effect in fat grafting that can be enhanced by hypoxia pre-treatment.

### hADSC-EVs promoted proliferation, migration and tube formation of human umbilical vein endothelial cells (HUVECs) in hypoxic conditions

**Figure 3:**
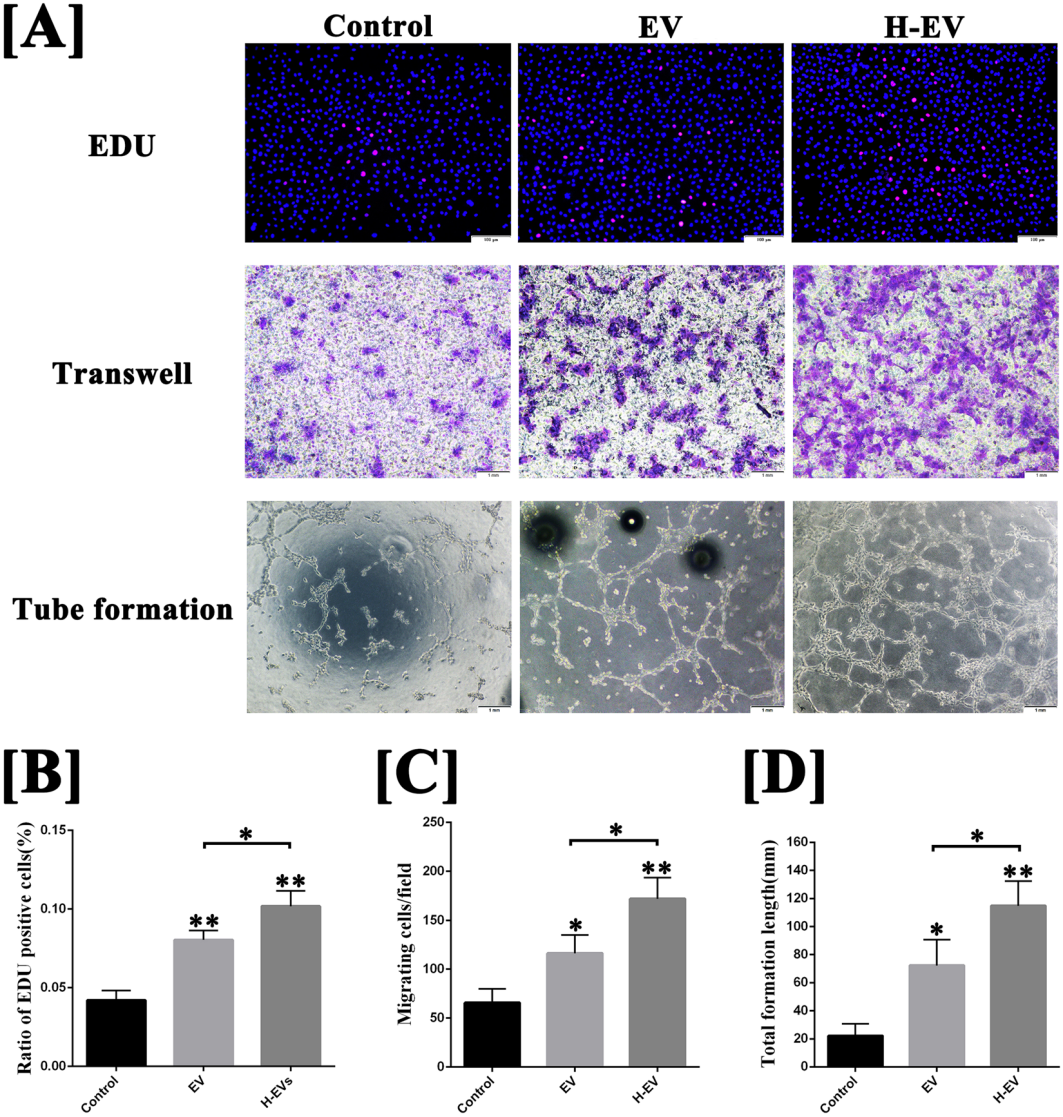
Effect of hADSC-EVs to HUVECs on the proliferation, migration and tube formation on Matrigel. (**A**) Image and (**B–D**) semi-quantitative EdU, transwell migration and tube formation assays of HUVECs with and without hypoxic hADSC-EVs or hADSC-EVs. The proliferation, migration and mean tube structure of HUVECs treated with hADSC-EVs or hypoxic hADSC-EVs were higher than those in the control group. Moreover, the ability of proliferation, migration and tube formation of HUVECs could be further enhanced by treatment with hypoxic hADSC-EVs (compared with normal hADSC-EVs). Data are presented as the mean ± SD (n = 3). *P < 0.05, **P < 0.01. Scale bar in EDU test: 100 μm. Scale bar in transwell and tube formation assays: 1 mm.

To further explore the molecular mechanisms of hADSC-EVs promoting angiogenesis, we conducted *in vitro* experiments in hypoxic conditions to mimic the angiogenesis in the early stage of fat grafting. As shown in Fig. 3A–D, the results of the EdU and transwell migration assays and tube formation on Matrigel in hypoxic condition showed that the proliferation, migration and tube formation of HUVECs were enhanced by hADSC-EVs (EDU: 8.0 ± 0.5%; Transwell: 116.3 ± 15.1/field; Tube formation: 72.3 ± 18.3 mm) or hypoxic hADSC-EVs (EDU: 10.2 ± 0.8%; Transwell: 172.0 ± 17.7/field; Tube formation: 114.8 ± 17.5 mm) treatment compared with the control group (EDU: 4.2 ± 0.5%; Transwell: 65.7 ± 11.7/field; Tube formation: 22.2 ± 8.5 mm) (P < 0.05). Moreover, those abilities could be further enhanced by treatment with hypoxic hADSC-EVs (P < 0.05).

### Hypoxia treatment increased the level of let-7 family in hADSC-EVs

**Figure 4:**
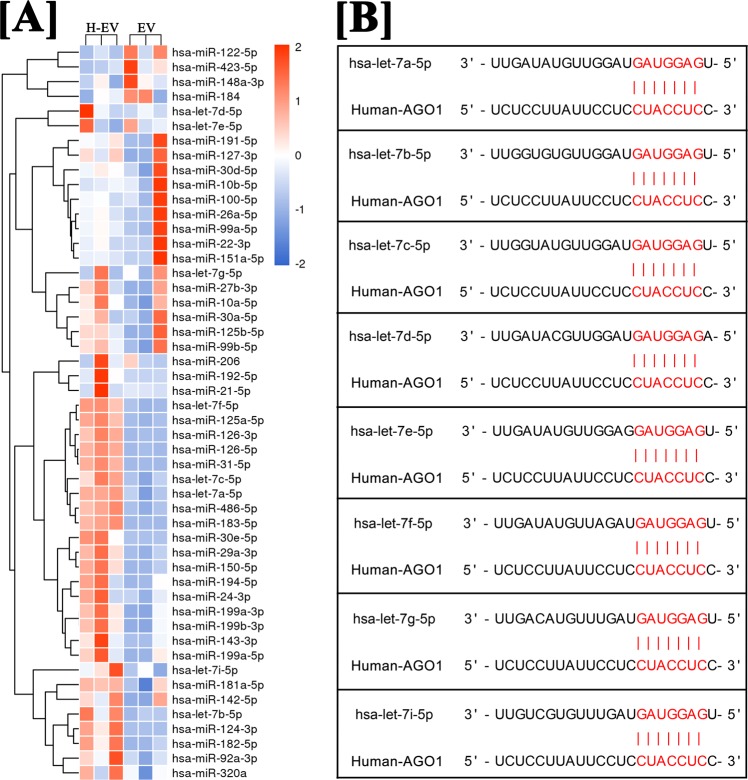
MicroRNA sequencing and targeting prediction. (**A**) Heatmap of the top 50 microRNA with the greatest difference between normal and hypoxic hADSC-EVs. (**B**) A list of predicted binding sites of let-7 family and AGO1.

As shown in Fig. 4A, eight kinds of let-7 were detected in microRNA sequencing of hADSC-EVs counted in top 50 microRNAs for the expression of transcripts per million (TPM), including let-7b-5p, let-7f-5p, let-7a-5p, let-7i-5p, let-7c-5p, let-7e-5p, let-7g-5p and let-7d-5p, and all of them were significantly upregulated in hypoxic hADSC-EVs for the expression of TPM. The analysis of microRNA targeting prediction showed that the argonaute 1/vascular endothelial growth factor (AGO1/VEGF) pathway was highly enriched in the gene ontology (GO) analysis, and eight kinds of microRNAs let-7 were all found to be directly targeted to AGO1of human beings but not to that of mouse (Fig. 4B).

### hADSC-EVs participated in the activity of proliferation, migration and tube formation in HUVECs via the let-7/AGO1/VEGF signalling pathway

**Figure 5:**
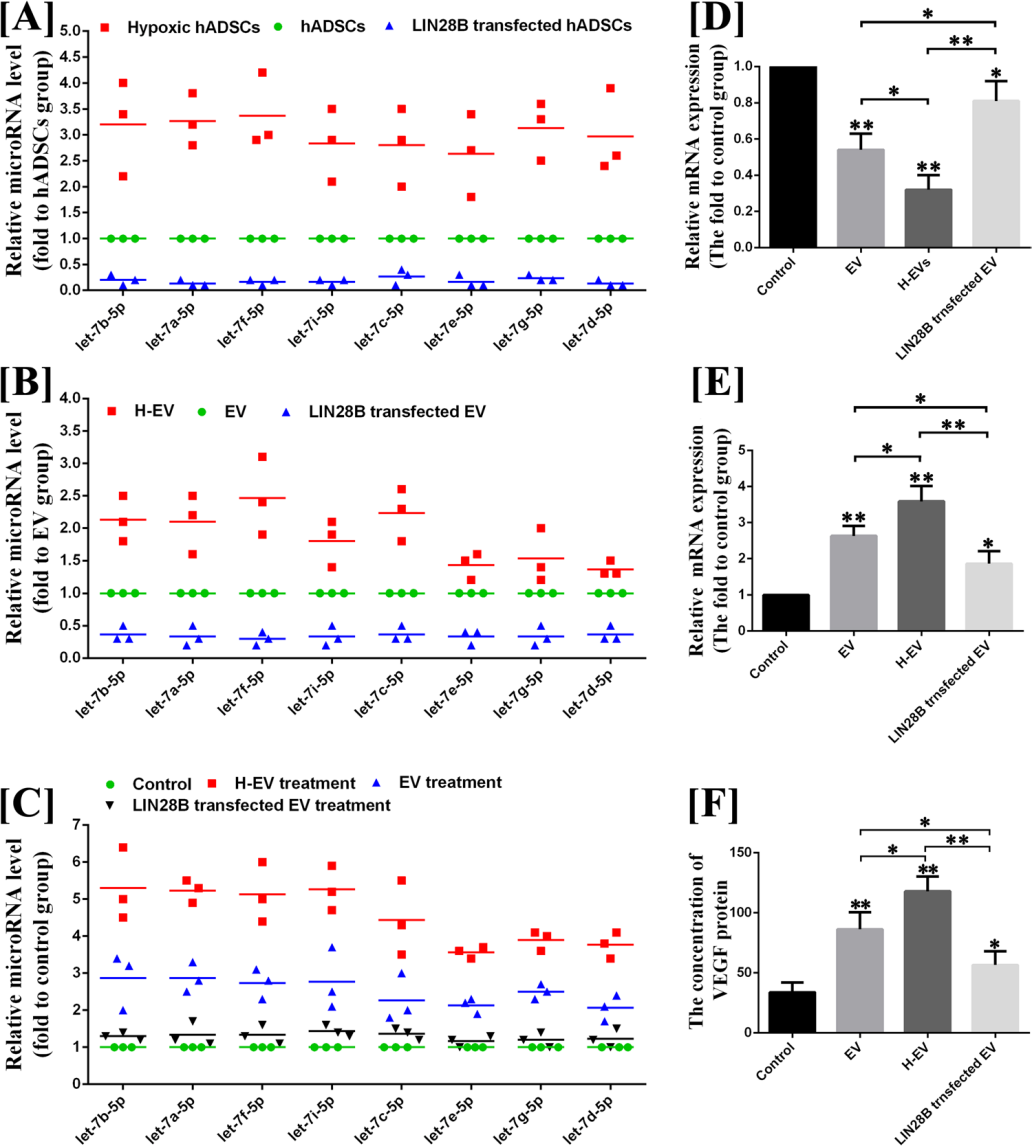
Let-7/AGO1/VEGF signalling pathway detection. (**A**) The results of qRT-PCR for let-7 families in hADSCs, hypoxic hADSCs and LIN28B transfected hADSCs. (**B**) The results of qRT-PCR for let-7 families in hADSC-EVs, hypoxic hADSC-EVs and LIN28B transfected hADSC-EVs. (**C**) The results of qRT-PCR for let-7 families in HUVECs treated by hADSC-EVs, hypoxic hADSC-EVs and LIN28B-hADSC-EVs. (**D**) The results of qRT-PCR for AGO1 in HUVECs treated by hADSC-EVs, hypoxic hADSC-EVs and LIN28B-hADSC-EVs. (**E**) The results of qRT-PCR for VEGF in HUVECs treated by hADSC-EVs, hypoxic hADSC-EVs and LIN28B-hADSC-EVs. (**F**) The results of ELISA assay for VEGF secretion of HUVECs treated by hADSC-EVs, hypoxic hADSC-EVs and LIN28B-hADSC-EVs. Data are presented as the mean ± SD (n = 3). *P < 0.05, **P < 0.01.

**Figure 6:**
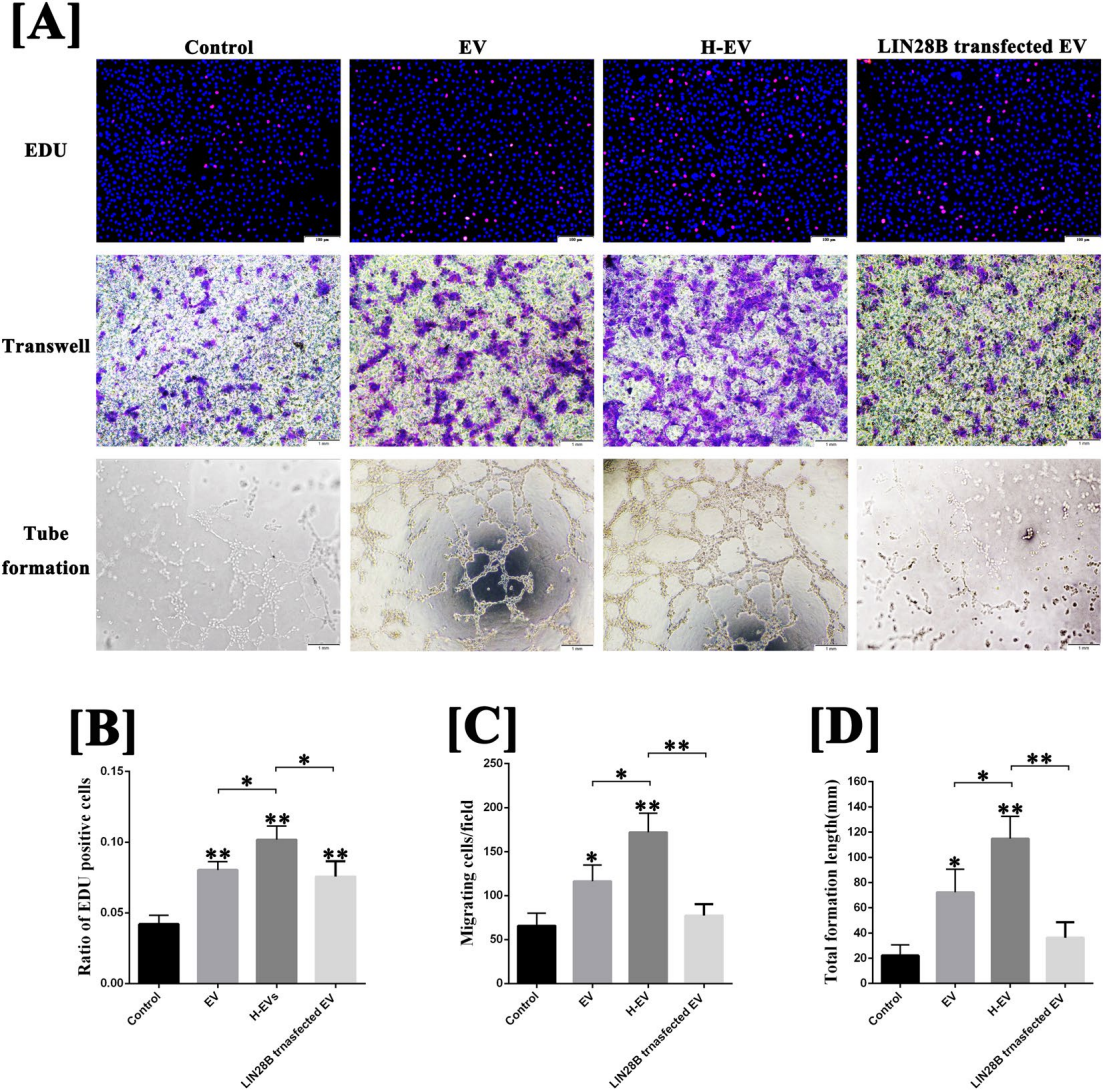
Effect of let-7-downregulated hADSC-EVs to HUVECs on the proliferation, migration and tube formation on Matrigel. (**A**) Image and (**B–D**) semi-quantitative EdU, transwell migration and tube formation assays of HUVECs with and without hADSC-EVs, hypoxic hADSC-EVs or LIN28B transfected hADSC-EVs. The results indicated that, compared with that of normal hADSC-EVs, blocking of let-7 impair the abilities of promoting migration and tube formation of hADSC-EVs, but has no significant effect on the ability to promote HUVEC proliferation. Data are presented as the mean ± SD (n = 3). *P < 0.05, **P < 0.01. Scale bar in EDU test: 100 μm. Scale bar in transwell and tube formation assays: 1 mm.

Because AGO1 is a well-known regulator of biological activity of vascular endothelial cells and VEGF mRNA transcription and protein synthesis^18–21^, we believe that let-7 could promote angiogenesis by targeting the AGO1/VEGF pathway. Thus, let-7 may play an important role in the pro-angiogenic molecular mechanism of hADSC-EVs. To validate this hypothesis, the LIN28B vector (an inhibitor to let-7 family) was transfected into hADSCs and cultured for 48 h to isolate the let-7 blocked EVs. The results of quantitative reverse transcription polymerase chain reaction (qRT-PCR) showed that the expression of all let-7 families in hADSCs was approximately 70–90% decreased after LIN28B transfection and in hADSC-EVs was approximately 50–80% decreased after LIN28B transfection. However, hypoxic treatment increased the expression of all let-7 families in both hADSCs and hADSC-EVs **(**Fig. 5A,B**)**. The let-7 families in hADSC-EVs were also involved in the regulation of the AGO1/VEGF pathway. In Fig. 5C–F, treatment with hADSC-EVs increased the expression of let-7 families **(**Fig. 5C**)**, inhibited the mRNA expression of AGO1 **(**Fig. 5D**)** and increased the mRNA expression and protein synthesis of VEGF **(**Fig. 5D,E**)** in HUVECs, compared with the control group (P < 0.05), and these effects were further enhanced by hypoxic hADSC-EV treatment (P < 0.05). However, the down- and up-regulating of AGO1 and VEGF in HUVECs were suppressed by LIN28B-hADSC-EV treatment **(**Fig. 5C–F**)**. In addition, the results of the EdU, transwell migration and tube formation assays in hypoxic conditions indicated that, compared with that of normal hADSC-EVs, blocking of let-7 impaired the abilities of promoting migration and tube formation of hADSC-EVs (P < 0.05) but has no significant effect on the ability to promote HUVECs proliferation (P > 0.05) **(**Fig. 6A–D**)**.

### hADSC-EVs promote the survival of fat grafts via the let-7/AGO1/VEGF signalling pathway

**Figure 7:**
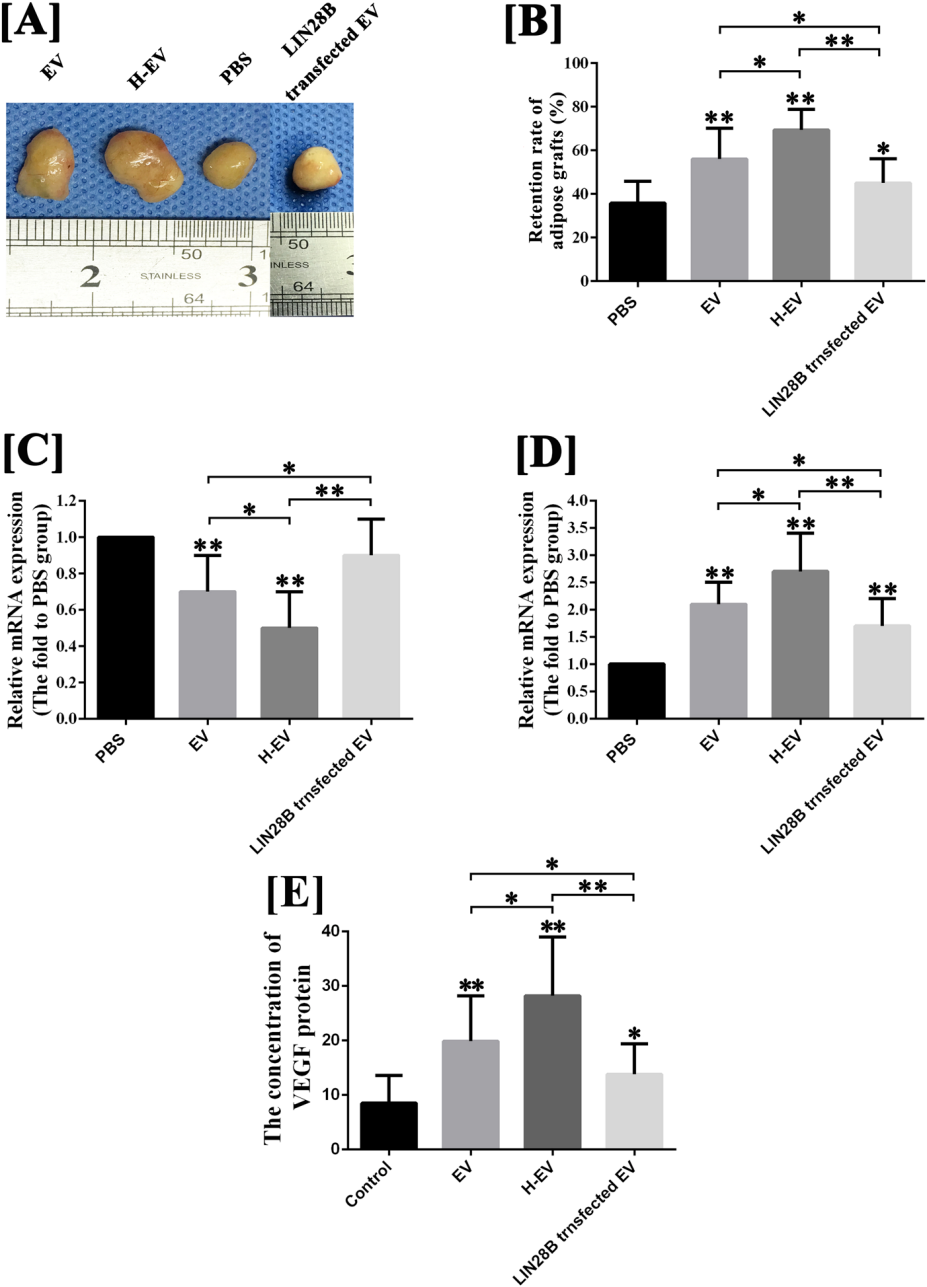
Detection of the let-7/AGO1/VEGF signalling pathway in a mouse model of fat grafting (n = 12). (**A**) The fat grafts harvested at post-grafted week 12. (**B**) The volume retention rate of fat grafts co-transplanted by LIN28B-hADSC-EVs was significantly decreased at post-grafting week 12, compared with grafts co-transplanted by normal hADSC-EVs, but was still higher than grafts co-transplanted by PBS. (**C**) The results of qRT-PCR for AGO1 mRNA expression of fat grafts at post-grafting day 3. (**D**) The results of qRT-PCR for VEGF mRNA expression of fat grafts at post-grafting day 3. (**E**) The results of ELISA for VEGF protein concentration of fat grafts at post-grafting day 3. Data are presented as the mean ± SD (n = 12). *P < 0.05, **P < 0.01.

**Figure 8:**
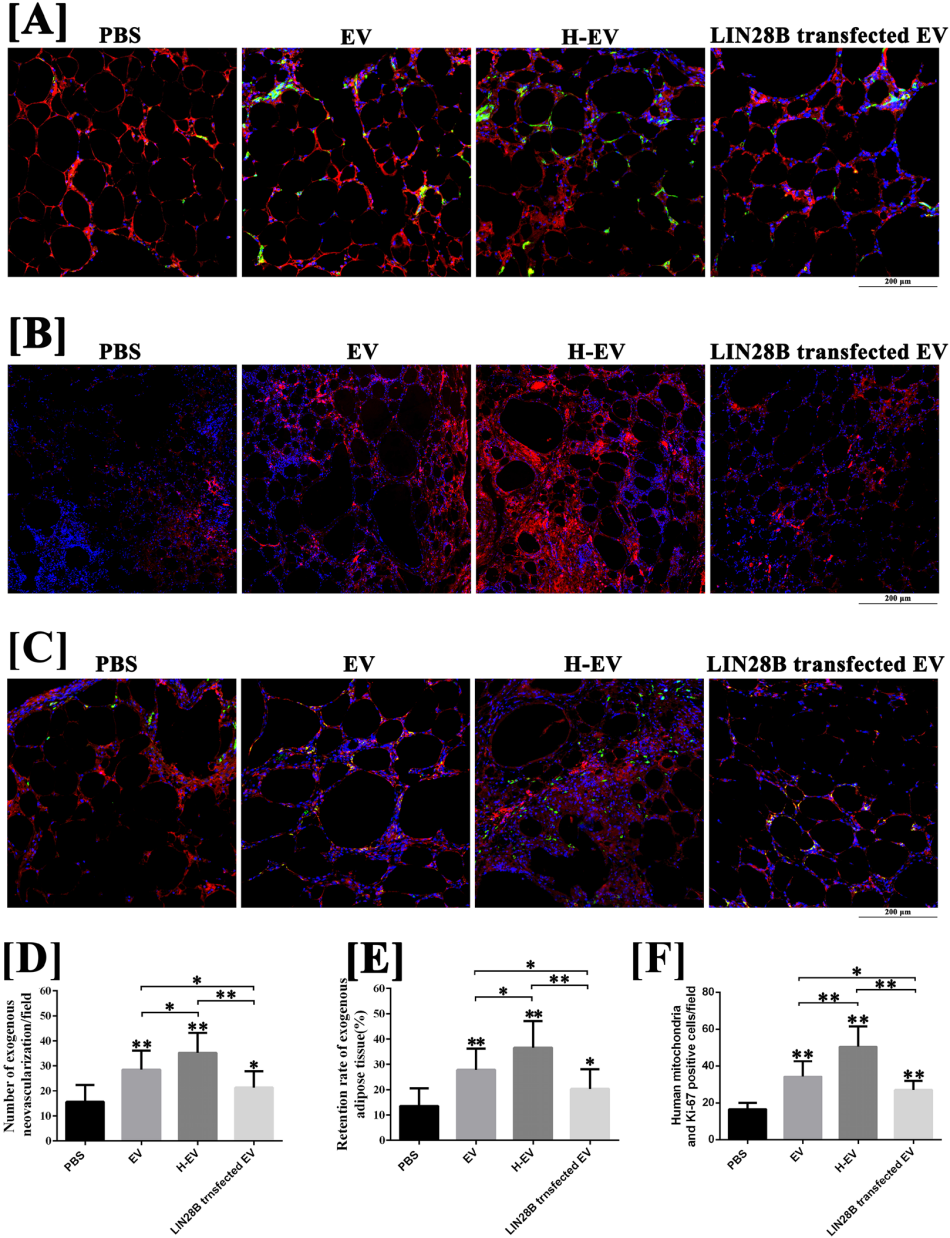
The histological analysis of fat grafts (n = 12). (**A,D**) The results of CD31(green)/human mitochondria immunofluorescence(red) staining indicated that, compared with grafts co-transplanted with hADSC-EVs, grafts co-transplanted with LIN28B-hADSC-EVs exhibited decreased exogenous neovascularisation counts at post-grafting week 4. however, the count of exogenous neovascularization of LIN28B-hADSC-EVs co-transplanted grafts were still higher than them in control grafts. (**B,E**) The results of immunofluorescence staining (human mitochondria) indicated that, compared with grafts co-transplanted with hADSC-EVs, grafts co-transplanted with LIN28B-hADSC-EVs performed and decreased retention rate of exogenous adipose tissue at post-grafting week 12. Of note, the retention rate of exogenous adipose tissue of LIN28B-hADSC-EVs co-transplanted grafts were still higher than those in control grafts. (**C,F**) The results of Ki-67(green)/human mitochondria immunofluorescence(red) staining indicated that, compared with control grafts, grafts co-transplanted with hADSC-EVs exhibited increased Ki-67 positive cells in grafts at post-grafting week 1 (P < 0.05), and grafts co-transplanted with hypoxic hADSC-EVs exhibited more Ki-67 positive cells in grafts compared with hADSC-EVs treated grafts at the same time point (P < 0.05). Moreover, decreased number of Ki-67 positive cells of LIN28B-hADSC-EVs co-transplanted grafts were detected compared with hADSC-EVs co-transplanted grafts (P < 0.05). However, the number of Ki-67 positive cells of LIN28B-hADSC-EVs co-transplanted grafts were still higher than that in control grafts (P < 0.05). Data are presented as the mean ± SD (n = 12). *P < 0.05, **P < 0.01. Scale bar: 200 μm.

As shown in Fig. 7A,B, the volume retention rate of fat grafts co-transplanted by LIN28B-hADSC-EVs was significantly decreased at post-grafting week 12, compared with grafts co-transplanted by normal hADSC-EVs, but was still higher than grafts co-transplanted by PBS. Furthermore, as shown in Fig. 7C–E, at post-grafting day 3, the results of qRT-PCR and enzyme-linked immunosorbent assay (ELISA) demonstrated that co-transplanting with hADSC-EVs could inhibit the mRNA expression of AGO1, and increase the mRNA expression and protein synthesis of VEGF in fat grafting, compared with the control group (P < 0.05), and these effects were further enhanced by hypoxic hADSC-EVs co-transplant (P < 0.05). However, the down- and upregulation of AGO1 and VEGF in fat grafts were suppressed by LIN28B-hADSC-EV co-transplant. Of note, the mRNA and protein level of VEGF in grafts co-transplanted with LIN28B-hADSC-EVs were still higher than those in control grafts (P < 0.05). For histological analysis, as shown in Fig. 8A,B and D,E, compared with grafts co-transplanted with hADSC-EVs, grafts co-transplanted with LIN28B-hADSC-EVs exhibited decreased exogenous neovascularisation count at post-grafting week 4 (P < 0.05) and decreased retention rate of exogenous adipose tissue at post-grafting week 12 (P < 0.05). Of note, the count of exogenous neovascularisation as well as retention rate of exogenous adipose tissue of LIN28B-hADSC-EVs co-transplanted grafts were still higher than them in control grafts (P < 0.05). In addition, according to the results of immunofluorescence staining for Ki-67 and human mitochondria (Fig. 8C,F), compared with control grafts, grafts co-transplanted with hADSC-EVs exhibited increased Ki-67 positive cells in grafts at post-grafting week 1 (P < 0.05), and grafts co-transplanted with hypoxic hADSC-EVs exhibited more Ki-67 positive cells in grafts compared with hADSC-EVs treated grafts at the same time point (P < 0.05). Moreover, decreased number of Ki-67 positive cells of LIN28B-hADSC-EVs co-transplanted grafts were detected compared with hADSC-EVs co-transplanted grafts (P < 0.05). However, the number of Ki-67 positive cells of LIN28B-hADSC-EVs co-transplanted grafts were still higher than that in control grafts (P < 0.05).

The above histological results suggest that, in the mouse model of fat grafting, let-7 in hADSC-EVs is involved in the regulation of exogenous angiogenesis and proliferation of transplanted cells, which influence volume retention.

## Discussion

Angiogenesis in the early stage of fat grafting depends on the contribution of both exogenous graft and the host. After transplantation, a part of graft will suffer necrosis due to the lack of blood supply and nutrition. In this way, only a few grafted adipose tissues can survive based on neovascularising^22^. Thus, approaches to promote angiogenesis in the early stage after transplantation might become a potential therapeutic strategy to improve the volume retention of fat grafts. MSC-EVs have been identified as important mediators of intracellular reactions and biologic effects, rather than merely being cell debris, in response to different stimuli or environmental circumstances^15^. In the present study, sufficient numbers of hADSC-EVs were collected and confirmed by surface markers, NanoSight and TEM. We suggested that hADSC-EVs possess pro-angiogenic capacity, including improving neovascularisation in ischaemic fat grafts *in vivo* and promoting proliferation, migration and tube formation of vascular endothelial cells in *in vitro* hypoxic conditions. Compared with other kinds of MSCs, ADSCs have a wide range of sources and easy access. Hence, co-transplantation of hADSC-EVs is also a feasible strategy for fat grafting.

By microRNA sequencing, multiple microRNAs in let-7 family were found to be enriched in hADSC-EVs. Let-7 has been found to be highly expressed in HUVECs and appears to play a key role in angiogenesis^23–25^. The effect of let-7 on angiogenesis was first identified^23^ by the Dicer and Drosha knockdown method (levels of let -7a, let-7b-5p, let-7c, let-7f-5p and let-7g were reduced by more than 30%). It has been demonstrated^23–25^ that the reduction of let-7b, let-7a and let-7f significantly impairs the sprouting of HUVEC blood vessels, but a detailed signalling network has not yet been determined. A recent study found that hypoxia-responsive microRNAs including let-7 target AGO1 to promote angiogenesis by de-suppress the mRNA transcription of VEGF^25,26^. Our findings suggested that hADSC-EVs deliver let-7 to vascular endothelial cells, target the let-7/AGO1/VEGF signalling pathway and promote the migration and tube formation. It is possible that hypoxic hADSC-EVs contributed more to pro-angiogenic effects because of the high levels of let-7 in EVs derived from human ADSCs cultured in hypoxic condition. However, according to the results of this study, the effect of hADSC-EVs promoting proliferation of vascular endothelial cells has no significant relationship with the delivery of let-7, which may be related to proteins or other microRNAs. According to the results, the effect of hADSC-EVs on the proliferation of transplanted cells *in vivo* may be related to the transmission of let-7, but we cannot rule out that this effect is indirectly derived by let-7, that is, let-7 promotes the early angiogenesis of grafts, and neovascularization directly promotes the proliferation of transplanted cells via the transport of nutrients. However, let-7 is not the sole influencing factor. Other angiogenic substances in the hADSC-EVs (proteins or other microRNAs) may also be involved in the angiogenesis or proliferation of fat grafts. In addition, we found through microRNA targeting prediction that let-7 can only target AGO1 mRNA of human and cannot target that of mice. This finding may be one of the main reasons why hADSC-EVs exert few effects on promoting host-derived angiogenesis in a nude mouse model of fat grafting.

The use of ADSC-EVs as an alternative to ADSCs engraftment may provide new perspectives to improve not only the survival of fat grafting but also therapy for various ischaemic diseases, such as heart stalk, limb ischaemia and diabetic wound. This approach has several advantages. First, ADSC-EVs is unlike the potential of ADSCs to undergo the risk of tumorigenesis as the result of their ability for multidirectional differentiation^27^. Second, EVs are more stable than ADSCs during storage and transportation^28,29^. Third, a large dose of ADSCs might cause pulmonary embolism after transfusion. ADSC-EVs are small and can bypass the lung when administered intravenously^30^.

As for the limitations of this study, we confirmed only that microRNA let-7 family in hypoxic hADSC-EVs promote angiogenesis by targeting the AGO1/VEGF pathway, but proteins and other microRNAs that may be involved in angiogenesis were not studied. In addition, there are several subtypes in the let-7 family, and whether those let-7 subtypes in hypoxic hADSC-EVs have different biological functions requires further study. These gaps in knowledge should be considered as the limitations of this study and will be addressed in our further research.

In summary, in the mouse model of fat grafting, our findings suggest that the survival of fat grafts can be improved by co-transplantation of hADSC-EVs and hypoxic hADSC-EVs, which enhance exogenous angiogenesis in an early stage post-transplantation. The mechanism of angiogenesis may be derived from the delivery of let-7 from hADSC-EVs to vascular endothelial cells targeting the let-7/AGO1/VEGF signalling pathway.

## Materials and methods

### Isolation and identification of human adipose-derived mesenchymal stem cells

For isolation of hADSCs, the following protocols were performed in accordance with the Declaration of Helsinki and approved by the Ethics Committee of the Second Affiliated Hospital of Nanchang University. Human subcutaneous adipose tissue was obtained from a healthy female donor, aged 24 years, who had undergone an abdominal liposuction bariatric procedure in the department of Plastic Surgery. Informed consent was obtained. Immediately after the liposuction procedure, the harvested lipoaspirates were centrifuged at 1,200 ×*g* for 3 min for purification. After centrifugation, the top oil layer and the bottom fluid layer were removed. The middle layer, which contains the purified lipoaspirate, was used in subsequent experiments. The entire harvesting procedure was performed at 25 °C. Ten millilitres of purified lipoaspirate were digested with an equal volume of 0.2% type I collagenase (Sigma, Silicon Valley, USA) in a constant temperature water bath shaker at 37 °C for approximately 1 h and filtrated by a 200-μm metal mesh. After filtration, the residues were centrifuged at 1,200 ×*g* for 3 min, and the supernatant was discarded. Precipitates were resuspended in culture medium and cultured at 37 °C in the presence of 5% CO_2_. The medium was first changed after 48 h and every 24 h afterward. The cells were passaged after reaching 90% confluency. The surface markers of third-generation cells, including CD49d, CD90, CD105, CD34 CD45, and CD73 (Abcam, London, UK) were detected by flow cytometry.

### Isolation and identification of EVs derived from human adipose-derived mesenchymal stem cells

hADSCs at passage 3 were cultured under hypoxia (5% oxygen) and normoxia (20% oxygen) until cells reached 70–80% confluency. The medium was then replaced with serum-free Dulbecco’s modified Eagle’s medium (DMEM)/F12 for 24 h to collect conditioned medium. Cell debris and apoptotic bodies were discarded after sequential centrifugations at 500 ×*g*, 3,000 ×*g* and 10,000 ×*g* for 5, 15 and 60 min, respectively. The supernatant was then ultra-centrifuged at 100,000 ×*g* at 4 °C for 1 h using a 45 Ti rotor (Beckman Coulter, Optimal L-80XP, USA). The remaining precipitate was resuspended in PBS and filtered with a 0.22-mm filter (Millipore, Billerica, MA, USA), then ultra-centrifuged at 100,000 ×*g* at 4 °C for 1 h. EVs were stored at −80 °C until further use. EVs obtained from both conditions were characterised using 80 kV TEM (HITACHI H-7000FA, Japan). The particle size distribution and concentrations of EVs were analysed by ZetaVIEW (Particle Metrix, ZetaVIEW S/N 17–315, Germany) and Nanoparticle Tracking Analysis software (ZetaVIEW 8.04.02). Antibodies against CD9, CD63, Alix, β-actin and calnexin (Abcam, London, UK) were used in western blotting. In addition, the supernatant of EV lysates was prepared, and the protein content was evaluated using a BCA Protein Assay Kit (Sigma, Silicon Valley, USA).

### Animal models

One hundred and eight six-week-old female BALB/c nude mice were purchased from the Animal Experimental Centre of Nanchang University. All animal experimental procedures were approved by the Committee of Animal Care of the Second Affiliated Hospital of Nanchang University and conducted according to the guidelines of the National Health and Medical Research Council of China. Mice were housed in an environment with temperatures ranging from 20 to 26 °C and humidity from 40 to 70%. A 12/12-hour light-dark cycle and an ad libitum chow diet were provided during the experiment. Mice were randomly divided into three groups (24 mice per group), anaesthetised with an intraperitoneal injection of pentobarbital (1%, 50 mg/kg). Human lipoaspirate (LPA) was transplanted into their scalps, using a 1.0-mL syringe with a 19-gauge needle, as follows: (1) PBS group, 300 μL LPA + 100 μL PBS; (2) EV group, 300 μL LPA + 100 μL hADSC-EVs (50 μg/mL); (3) H-EV group, 300 μL LPA + 100 μL hypoxic hADSC-EVs (50 μg/mL). After transplantation, the skin incision was closed with a 6–0 non-absorbable suture. Grafts from each group were harvested at day 3 and weeks 4 and 12 post-transplantation (12 mice per group per time point). The volume of grafts at post-grafting week 12 were measured by drainage. The retention rate (measured volume/initial volume) of each fat graft was calculated.

### Immunofluorescence (IF; CD31, Ki-67 and human mitochondria) staining

Harvested grafts were fixed in 10% buffered formalin, embedded in paraffin, sectioned into 5-µm slices and subjected to IF staining, and sections were deparaffinised and incubated overnight at 4 °C with anti-CD31(1:50, Abcam, London, UK), anti-Ki-67 (1:500, Abcam, London, UK) and anti-human mitochondria (1:300, Abcam, London, UK) primary antibodies. The sections were placed in PBS and washed three times on a decolourising shaker, for 5 min each time. Secondary antibodies, fluorescein isothiocyanate (FITC)-conjugated goat anti-rabbit or CY3-conjugated goat anti-mouse (Bioss, Beijing, China) were then added for 50 min at room temperature in the dark. Nuclei were stained with 4,6-diamino-2-phenylindole (DAPI, Sigma-Aldrich, Silicon Valley, USA), and the slides were examined in a fluorescence microscope. To address angiogenesis of early stage post-grafting, we respectively calculated the average number of both exogenous (CD31^+^, human mitochondria^+^) and host-derived vessels (CD31^+^, human mitochondria^−^) from five random fields (×400) of each section of post-grafting week 4. In order to assess the retention rate of exogenous fat grafts, the percentage of area (human mitochondria^+^/DAPI^+^) from the five random fields (×400) of each section of post-grafting week 12 was calculated by Image-pro Plus 6.0 (Media Cybernetics, Inc., Rockville, MD, USA).

### EdU proliferation assay

To test the effect of hADSC-EVs on the proliferation of HUVECs in hypoxic conditions (5% oxygen), 100 μL of HUVECs suspension was added to a 96-well plate at 1×10^4^ cells per well. After complete cell adherence, HUVECs were cultured in foetal bovine serum (FBS)-free medium with 50 μg/mL hADSC-EVs obtained from hypoxic or normoxic conditions for 24 h, and HUVECs cultured in FBS-free medium were considered the control group. An EdU kit (RiboBio, Guangzhou, China) was employed for the cell proliferation assay. Briefly, cells were incubated with 50 μL of EdU medium for 2 h and, then, fixed and stained according to the manufacturer’s protocol. Five fields (×200) of each sample were randomly photographed in a fluorescence microscope, and the average numbers of total (blue) and proliferating (red) cells were calculated. The cell proliferation rate was calculated as follows: cell proliferation rate = the number of proliferating cells/the total number of cells × 100%.

### Migration assay

The migration assay was performed in a transwell system (Corning, New York, USA) of hypoxic condition (5% oxygen). HUVECs were resuspended in FBS-free medium at 2×10^5^/mL, and 200 μL of this suspension were seeded in the upper chamber; 500 μL of FBS-free medium containing hADSC-EVs or hypoxic hADSC-EVs (both at 50 μg/mL) were added to the lower chamber. FBS-free medium was used as control. Twenty-four hours later, the residual cells in the upper chamber were gently wiped off using sterile cotton swabs. Cells were fixed in 4% paraformaldehyde, stained with crystal violet, washed with distilled water, then observed and photographed in phase contrast microscope (×200) to determine the portion of migrated cells in each group.

### Tube formation assay

Matrigel (Corning, New York, USA) was transferred to 24-well culture plates and incubated at 37 °C for 30 min for coagulation. HUVECs were seeded at 5×10^4^ per well. Cells of experiment groups were supplemented with 500 μL of endothelial cell basal medium (ScienCell, San Diego, California, USA) containing 50 μg/mL of hADSC-EVs or hypoxic hADSC-EVs, and cells of the control group were supplemented with an equal volume of endothelial cell basal medium. After incubation for 12 h at 37 °C in hypoxic condition (5% oxygen), tube structure formation was observed and photographed by phase contrast microscopy (×200). The length of tube structure was measured by Image-Pro plus 6.0 (Media Cybernetics, Inc., Rockville, MD, USA).

### ELISA for VEGF

HUVECs were seeded at 5×10^4^ per 24-well and incubated at 37 °C for two days until cell density reached 80%. For two experiment group, replacing the complete medium with serum-free medium containing 50 μg/mL of hADSC-EVs or hypoxic hADSC-EVs. For control group, replacing the complete medium with serum-free medium containing. After 24 h incubated at 37 °C in hypoxic conditions (5% oxygen), the supernatant of each group was collected and submitted to ELISA assay for the concentration of VEGF protein by Human VEGF ELISA Kit (Abcam, London, UK).

### MicroRNA sequencing

To further explore the underlying mechanism of the pro-angiogenic effect induced by hADSC-EVs, microRNAs from hADSC-EVs were sequenced for three independent experiments. After total RNA extraction with TRIzol, RNA molecules in a size range of 18–30 nt were enriched by polyacrylamide gel electrophoresis (PAGE), and the 3’ adapters were added, and 36–44 nt-long RNAs were enriched. The 5′ adapters were then ligated to the RNAs. The ligation products were reverse-transcribed and PCR-amplified, and the 140–160 bp PCR products were enriched to generate a cDNA library and sequenced using Illumina HiSeqTM 2500 (Illumina, USA) by Gene Denovo Biotechnology Co. (Guangzhou, China). The microRNAs of hADSC-EVs were compared with the human microRNA sequences in miRBase by Bowtie software (version 1.1.2). To further reveal the possible roles of these microRNAs, we predicted their target genes and their functions using TargetScan (available at http://www.targetscan.org/) and gene ontology (GO) analysis.

### Blocking let-7 microRNA biogenesis by LIN28B transfection

LIN28B was cloned into pFLAG-CMV2 vector (Sigma, Silicon Valley, USA), as previously described^18^ and then transfected into hADSCs using Lipofectamine 3000 (Life Technologies) according to the manufacturer’s instructions. Six hours after transfection, complete medium was replaced. After 72 h in culture, the cells were collected for EV isolation. Quantitative Reverse-Transcriptase Polymerase Chain Reaction (qRT-PCR) was used to assess the expression of let-7b-5p, let-7f-5p, let-7a-5p, let-7i-5p, let-7c-5p, let-7e-5p, let-7g-5p, let-7d-5p in normal, hypoxic and LIN28B transfected hADSCs and hADSC-EVs. Primer sequences are listed in Table 1.

**Table 1:** Primer Sequences for Quantitative Reverse-Transcriptase Polymerase Chain Reaction.

Genies	Reverse Transcription Primer (5′-3′)	Forward Primer (5′-3′)	Reverse Primer (5′-3′)
**hsa-let-7i-5p**	CTCAACTGGTGTCGTGGAGTCGGCAATTCAGTTGAGAACAGCAC	ACACTCCAGCTGGGTGAGGTAGTAGTTTGT	TGGTGTCGTGGAGTCG
**hsa-let-7b-5p**	CTCAACTGGTGTCGTGGAGTCGGCAATTCAGTTGAGAACCACAC	ACACTCCAGCTGGGTGAGGTAGTAGGTTGT	TGGTGTCGTGGAGTCG
**hsa-let-7f-5p**	CTCAACTGGTGTCGTGGAGTCGGCAATTCAGTTGAGAACTATAC	ACACTCCAGCTGGGTGAGGTAGTAGATTGT	TGGTGTCGTGGAGTCG
**hsa-let-7a-5p**	CTCAACTGGTGTCGTGGAGTCGGCAATTCAGTTGAGAACTATAC	ACACTCCAGCTGGGTGAGGTAGTAGGTTGT	TGGTGTCGTGGAGTCG
**hsa-let-7c-5p**	CTCAACTGGTGTCGTGGAGTCGGCAATTCAGTTGAGAACCATAC	ACACTCCAGCTGGGTGAGGTAGTAGGTTGT	TGGTGTCGTGGAGTCG
**hsa-1et-7g-5p**	CTCAACTGGTGTCGTGGAGTCGGCAATTCAGTTGAGAACTGTAC	ACACTCCAGCTGGGTGAGGTAGTAGTTTGT	TGGTGTCGTGGAGTCG
**has-let-7e-5p**	CTCAACTGGTGTCGTGGAGTCGGCAATTCAGTTGAGAACTATAC	ACACTCCAGCTGGGTGAGGTAGGAGGTTGT	TGGTGTCGTGGAGTCG
**hsa-1et-7d-5p**	CTCAACTGGTGTCGTGGAGTCGGCAATTCAGTTGAGAACTATGC	ACACTCCAGCTGGGAGAGGTAGTAGGTTGC	TGGTGTCGTGGAGTCG
**β-actin**		CACCCAGCACAATGAAGATCAAGAT	CCAGTTTTTAAATCCTGAGTCAAGC
**AGO1**		CGAGGTCTGGTTCGGCTTTC	TGTAAGGGGAATGTCTGATGGC
**hVEGF-a**		GGAGGGCAGAATCATCACGA	GCTCATCTCTCCTATGTGCTGG

### Assay of let-7-AGO1-VEGF signalling pathway ***in vitro***

FBS-free medium with 50 μg/mL of normal, hypoxic or LIN28B transfected hADSC-EVs were co-cultured with HUVECs for 24 h in hypoxic conditions (5% oxygen). HUVECs cultured in FBS-free medium were employed as the control group. The expression of let-7b-5p, let-7f-5p, let-7a-5p, let-7i-5p, let-7c-5p, let-7e-5p, let-7g-5p, let-7d-5p and the RNA levels of AGO1 and VEGF in HUVECs were assessed by qRT-PCR. Primer sequences are listed in Table 1. EdU proliferation assay, transwell migration assay, and tube formation on Matrigel were also performed in hypoxic condition to evaluate the proliferation, migration and tube formation of HUVECs.

### Assay of let-7-AGO1-VEGF signalling pathway ***in vivo***

One hundred microlitres of LIN28B transfected hADSC-EVs (50 μg/mL) were co-transplanted with 300 μL of LPA into the scalps of 36 nude mice. The grafts at post-grafting day 3 were harvested to extract RNA with TRIzol and to extract protein with radioimmunoprecipitation assay (RIPA) lysate after homogenisation, then submitted to qRT-PCR for AGO1 and VEGF and ELISA for VEGF. The grafts at post-grafting week 4 and 12 were harvested and volume measured. Harvested grafts were fixed in 10% buffered formalin, embedded in paraffin sectioned into 5-µm slices, and subjected to CD31-human mitochondria IF staining as previously described. The angiogenesis and the retention rate of exogenous fat grafts were also evaluated.

### Statistical analysis

All statistical analyses were performed in GraphPad Prism (version 7.0, GraphPad Software Inc., La Jolla, CA). For *in vivo* experiments, all values are expressed as the mean ± standard deviation (SD) from 12 samples per group per time point. For *in vitro* experiments, all values are expressed as the mean ± SD from three independent experiments. Statistical significance of differences was evaluated by Student’s t-test or one-way analysis of variance (ANOVA). Data with a P value < 0.05 were considered significant. Detailed evaluation of all the samples was performed by two of the authors, who were blinded to the information regarding sample identity.

## Declarations

### Ethics approval and consent to participate

The subcutaneous adipose tissue for isolating human adipose-derived stem cells were obtained from healthy female donor who had undergone an abdominal liposuction bariatric procedure in department of plastic surgery, the Second Affiliated Hospital of Nanchang University; informed consent was obtained. No organs/tissues were procured from prisoners. The protocols above were performed in accordance with the Declaration of Helsinki and approved by the Ethics Committee of the Second Affiliated Hospital of Nanchang University.

All animal experimental procedures were approved by the Committee of Animal Care of the Second Affiliated Hospital of Nanchang University and conducted according to the guidelines of National Health and Medical Research Council of China.

## Consent for publication

All authors declare their support for the publication and its contents.

## Supplementary information

Supplementary Dataset 1.

## Data Availability

All data supporting the conclusions of this article are included within the article and its supplementary files.

## References

[1] Del Vecchio DA (2018). Clinical Implications of Gluteal Fat Graft Migration. Plast. Reconstr. Surg..

[2] Canizares O (2017). The Effect of Processing Technique On Fat Graft Survival. Plast. Reconstr. Surg..

[3] Roca E (2019). Abdominal Fat Graft for Sellar Reconstruction: Retrospective Outcomes Review and Technical Note. Operative Neurosurgery..

[4] Pu LLQ (2016). Mechanisms of Fat Graft Survival. Ann. Plas. Surg..

[5] Luck J, Smith OJ, Mosahebi A (2017). A Systematic Review of Autologous Platelet-Rich Plasma and Fat Graft Preparation Methods. Plastic and Reconstructive Surgery - Global Open..

[6] Yoshimura K (2008). Cell-Assisted Lipotransfer for Cosmetic Breast Augmentation: Supportive Use of Adipose-Derived Stem/Stromal Cells. Aesthet. Plast. Surg..

[7] Laloze J (2018). Cell-Assisted Lipotransfer: Friend Or Foe in Fat Grafting? Systematic Review and Meta-Analysis. J. Tissue Eng. Regen. M..

[8] Laloze J (2017). Cell-Assisted Lipotransfer: Current Concepts. Annales de Chirurgie Plastique Esthétique..

[9] Kabataş S (2018). Wharton’s Jelly-Derived Mesenchymal Stem Cell Transplantation in a Patient with Hypoxic-Ischemic Encephalopathy. Cell Transplant..

[10] Hawkins KE (2018). Embryonic Stem Cell-Derived Mesenchymal Stem Cells (MSCs) Have a Superior Neuroprotective Capacity Over Fetal MSCs in the Hypoxic-Ischemic Mouse Brain. Stem Cells Transl Med..

[11] Hong, K. Y. *et al*. The Fate of the Adipose-Derived Stromal Cells During Angiogenesis and Adipogenesis After Cell-Assisted Lipotransfer. *Plast. Reconstr. Surg*. **1** (2018). 10.1097/PRS.0000000000004021 29036025

[12] Tkach M, Thery C (2016). Communication by Extracellular Vesicles: Where we are and Where we Need to Go. Cell..

[13] Wang X (2016). Hsp20-Mediated Activation of Exosome Biogenesis in Cardiomyocytes Improves Cardiac Function and Angiogenesis in Diabetic Mice. Diabetes..

[14] Liu Y (2014). Microvesicles Secreted From Human Multiple Myeloma Cells Promote Angiogenesis. Acta Pharmacol. Sin..

[15] Zhang H (2012). Microvesicles Derived from Human Umbilical Cord Mesenchymal Stem Cells Stimulated by Hypoxia Promote Angiogenesis Both *in Vitro* and *in Vivo*. Stem Cells Dev..

[16] Cai Q (2018). Plants Send Small RNAs in Extracellular Vesicles to Fungal Pathogen to Silence Virulence Genes. Science (New York, N.Y.)..

[17] Mohr A, Mott J (2015). Overview of MicroRNA Biology. Semin. Liver Dis..

[18] Piskounova E (2011). Lin28A and Lin28B Inhibit Let-7 microRNA Biogenesis by Distinct Mechanisms. Cell..

[19] Yao J (2019). Extracellular Vesicles Derived From Human Umbilical Cord Mesenchymal Stem Cells Alleviate Rat Hepatic Ischemia-Reperfusion Injury by Suppressing Oxidative Stress and Neutrophil Inflammatory Response. The FASEB Journal..

[20] Peng K, Liu Y, Li Y, Yen BL, Yen M (2017). Extracellular Matrix Protein Laminin Enhances Mesenchymal Stem Cell (MSC) Paracrine Function through αvβ3/CD61 Integrin to Reduce Cardiomyocyte Apoptosis. J. Cell. Mol. Med..

[21] Zhang B (2015). HucMSC‐Exosome Mediated‐Wnt4 Signaling is Required for Cutaneous Wound Healing. Stem Cells..

[22] Mashiko T, Yoshimura K (2015). How Does Fat Survive and Remodel After Grafting?. Clin. Plast. Surg..

[23] Kuehbacher A, Urbich C, Zeiher AM, Dimmeler S (2007). Role of Dicer and Drosha for Endothelial MicroRNA Expression and Angiogenesis. Circ. Res..

[24] Bao M (2013). Let-7 in Cardiovascular Diseases, Heart Development and Cardiovascular Differentiation from Stem Cells. Int. J. Mol. Sci..

[25] Chen, Z. *et al*. Hypoxia-Responsive miRNAs Target Argonaute 1 to Promote Angiogenesis. *The Journal of Clinical Investigation*. 1057–1067 (2013). 10.1172/JCI65344 PMC3582133 23426184

[26] Bae O (2013). Oxidative Stress–Mediated Thrombospondin-2 Upregulation Impairs Bone Marrow–Derived Angiogenic Cell Function in Diabetes Mellitus. Arteriosclerosis, Thrombosis, and Vascular Biology..

[27] Liew A, O’Brien T (2012). Therapeutic Potential for Mesenchymal Stem Cell Transplantation in Critical Limb Ischemia. Stem Cell Res. Ther..

[28] Ge Q (2014). MiRNA in Plasma Exosome is Stable under Different Storage Conditions. Molecules..

[29] Maroto R (2017). Effects of Storage Temperature On Airway Exosome Integrity for Diagnostic and Functional Analyses. Journal of Extracellular Vesicles..

[30] DG P, MF P (2017). Concise Review: MSC-Derived Exosomes for Cell-Free Therapy. Stem Cells..

