# Supplementary material for: Extracellular vesicles derived from human adipose-derived stem cells promote the exogenous angiogenesis of fat grafts via the let-7/AGO1/VEGF signalling pathway
Source: Sci Rep. 2020 Mar 24;10:5313. doi: 10.1038/s41598-020-62140-6 (PMC7093512; doi:10.1038/s41598-020-62140-6)
Supplement: Supplementary file 1 — Supplementary Dataset 1. [file 41598_2020_62140_MOESM1_ESM.pdf]

Supplementary Materials

Extracellular vesicles derived from human adipose-derived stem cells promote the exogenous angiogenesis of fat grafts via the let-7/AGO1/VEGF signalling pathway

Yuan-zheng Zhu[+], Jing-Zhang[+], Xuan Hu, Zhao-hui Wang, Shu Wu, Yang-yan Yi [*]

Affiliations:

Department of Plastic Surgery, The Second Affiliated Hospital of Nanchang University, NO.1 of Minde Road of Nanchang, Jiangxi, 330006, P. R. China

Corresponding Authors:

Yang-yan Yi, Department of Plastic Surgery, The Second Affiliated Hospital of Nanchang University, 1 Minde Road of Nanchang, Jiangxi 330006, P. R. China

Email: yyy0218@126.com

Yuan-zheng Zhu and Jing Zhang contributed equally and are co-first authors.

Author's Email

Corresponding Authors: Yang-yan Yi, Email: yyy0218@126.com

Co-first author: Yuan-zheng Zhu, Email: 495684001@qq.com

Co-first author: Jing Zhang, Email: 2272013378@qq.com

Co-author: Xuan Hu, Email: 740202779@qq.com

Co-author: Zhao-hui Wang, Email: wangzhaohui85@163.com

Co-author: Shu Wu, Email: beata54@163.com

Materials and Methods

1. Tracing hADSC-EVs *in vivo*

hADSC-EVs were incubated with PKH26 for 20 min, according to the manufacturer instructions (Sigma-Aldrich, Silicon Valley， USA), and, then, centrifuged at 100,000 ×*g* for 60 min at 4°C to remove the supernatant. One hundred microliters of 50 µg/mL PKH26-labeled hADSC-EVs were subcutaneously co-transplanted with 0.3 mL free fat into the scalp of nude mice. The graft was removed 3 days after transplantation and fixed in 4% paraformaldehyde. Fixed tissues were frozen, sliced, and incubated overnight, at 4°C, with anti-CD31 primary antibody (1:50, Abcam, London, UK). CY3-conjugated goat anti-rabbit (Servicebio, Wuhan, China) was used as secondary antibody. Nuclei were stained with 4,6-diamino-2-phenylindole (DAPI, Sigma-Aldrich, Silicon Valley， USA), and the slides were examined in a fluorescence microscope (Olympus, Shinjuku City, Tokyo， Japan).

2. Tracing hADSC-EVs *in vtro*

Human umbilical vein endothelial cells (HUVECs), purchased from ScienCell Research Laboratories (Carlsbad, CA, USA), were inoculated into FBS-free medium and placed at 37°C in the presence of 5% CO_2 for cell attachment. After incubation with 50 µg/mL PKH26-labeled hADSC-EVs for 24 h, cells were washed twice with PBS, fixed in 4% paraformaldehyde, and stained with DAPI. The uptake of EVs was observed using confocal microscopy (Leica, SP5, Germany).

3. LIN28B transfection

LIN28B was cloned into pFLAG-CMV2 vector (Sigma, Silicon Valley, USA) and then transfected into hADSCs using Lipofectamine 3000 (Life Technologies) according to the manufacturer's instructions. Six hours after transfection, complete medium was replaced. After 72 h in culture, the cells were cultured in serum-free medium under 5% oxygen and 20% oxygen for EV isolation. Quantitative Reverse-Transcriptase Polymerase Chain Reaction (qRT-PCR) was used to assess the expression of let-7b-5p, let-7f-5p, let-7a-5p, let-7i-5p, let-7c-5p, let-7e-5p, let-7g-5p, let-7d-5p in LIN28B

transfected hADSCs and hADSC-EVs collected in both normoxic and hypoxic condition.

Supplementary Text

Supplementary Text S1: hADSCs-EVs tracing results *in vivo*

After co-transplantation of free fat with PKH26-labeled hADSC-EVs for 3 days, the results of fluorescence microscopy showed that red fluorescence was visible on the nucleus and partially overlapped with the green fluorescence (CD31), suggesting that some hADSC-EVs were internalized by vascular endothelial cells *in vivo* **(Figure S1)**.

Supplementary Text S2: hADSCs-EVs tracing results *in vitro*

In order to determine whether hADSC-EVs can be captured by HUVECs, we performed a hADSC-EVs tracing experiment. After incubation of HUVECs with PKH26-labeled (red) hADSC-EVs for 24 h, the results of confocal microscopy showed that red fluorescence was visible around the nucleus of HUVECs, suggesting that hADSC-EVs can enter the cytoplasm of HUVECs and distribute around the nucleus **(Figure S2)**.

Supplementary Text S3: Hypoxia cannot increase the level of let-7 family in LIN28B transfected hADSCs and hADSC-EVs

LIN28B transfected hADSCs were subjected to both normoxic and hypoxic condition and detect the expression levels of let-7 family of both hADSCs and hADSC-EVs. The results indicated that hypoxic pretreatment cannot increase the let-7 family level in LIN28B transfected hADSCs and hADSC-EVs, compared with that cultured in normoxic condition **(Figure S3-4)**.

Supplementary Figures

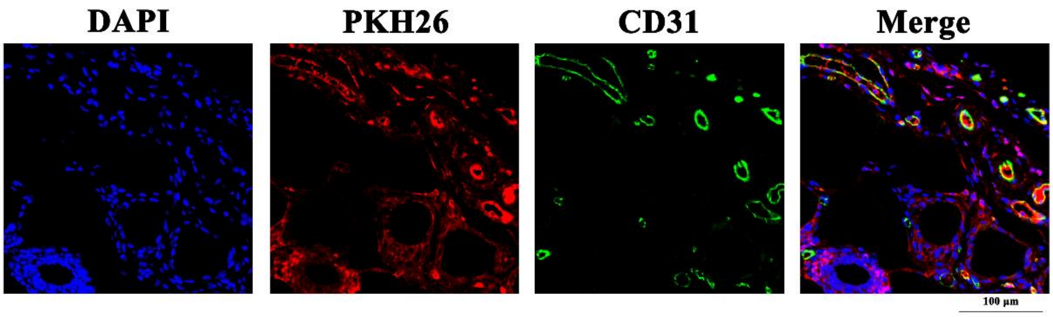

Figure S1: In vivo uptake of hADSC-EVs by vascular endothelial cells. The results of fluorescence microscopy showed that red fluorescence was visible on the nucleus and partially overlapped with the green fluorescence (CD31), suggesting that part of hADSC-EVs were internalized by vascular endothelial cells in fat grafts. Scale bar: 100 μm.

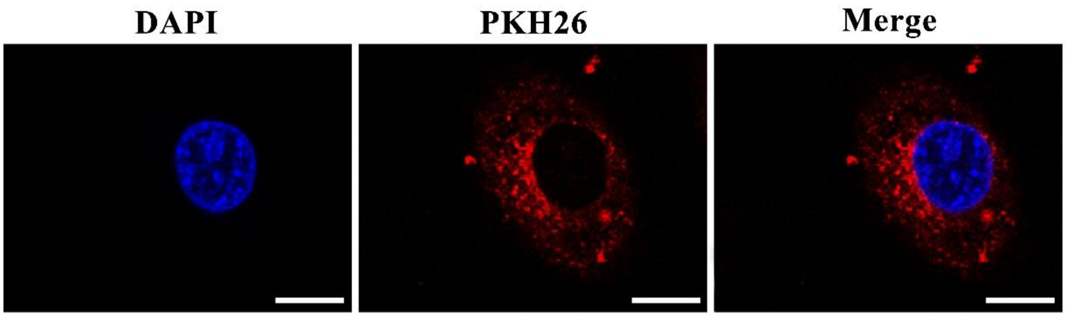

Figure S2: In vitro uptake of hADSC-EVs by HUVECs. Red fluorescence is visible around the nucleus of HUVECs. Scale bar: 10 μm.

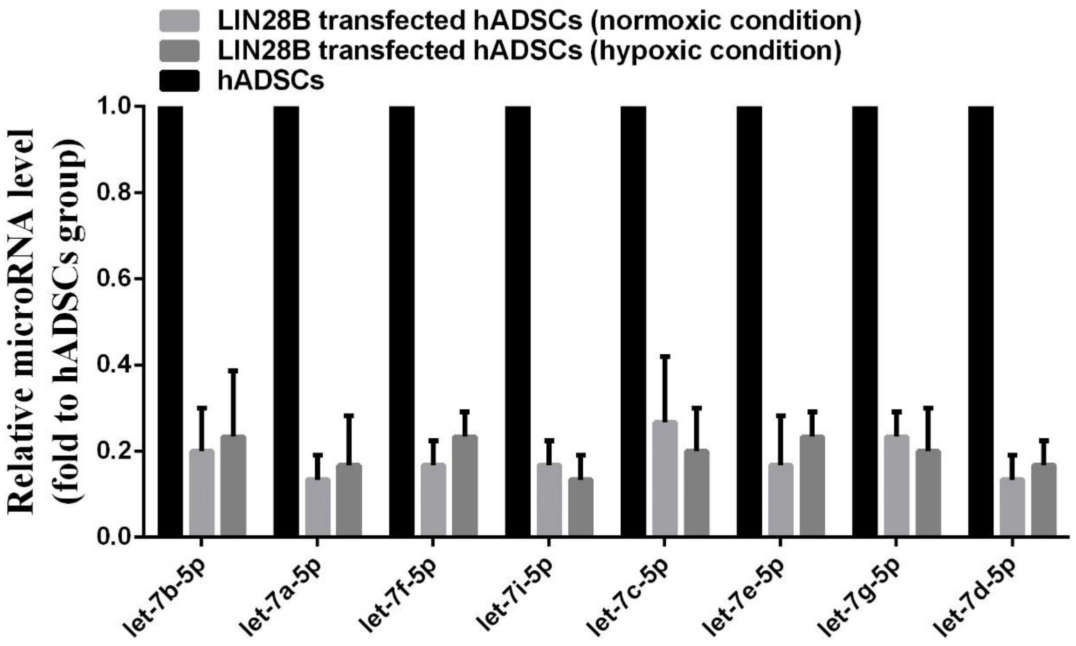

Figure S3: Hypoxia cannot increase the level of let-7 family in LIN28B transfected hADSCs

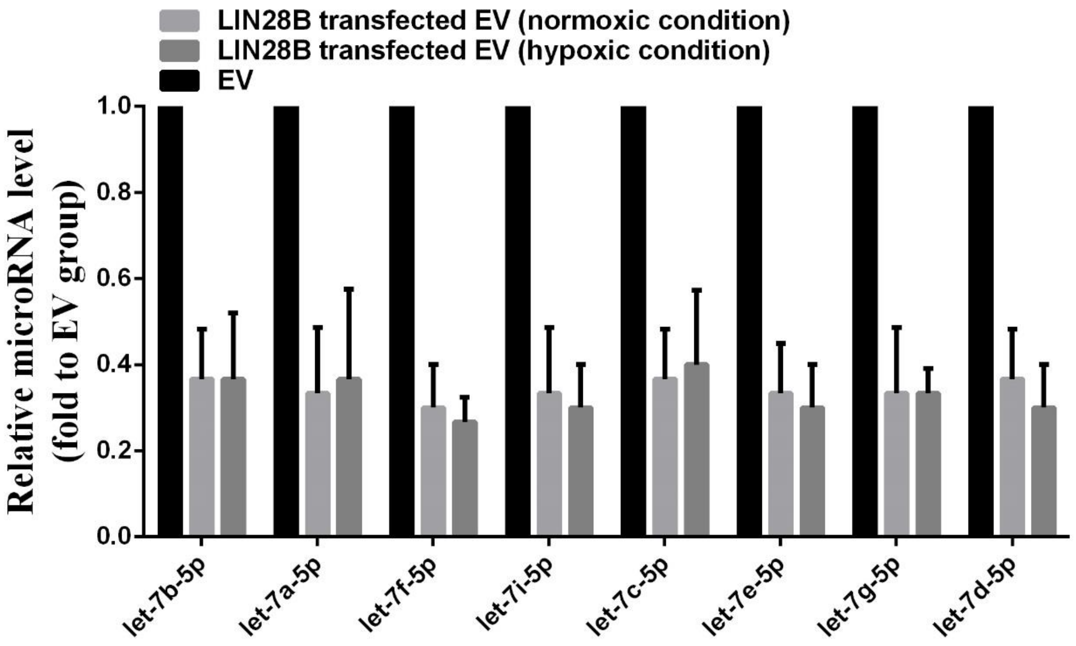

Figure S4: Hypoxia cannot increase the level of let-7 family in LIN28B transfected hADSC-EVs
